# The Variation in Outcomes of Septic Patients: A Dual-Centre Comparative Study

**DOI:** 10.7759/cureus.30677

**Published:** 2022-10-25

**Authors:** Hassan Baig, Tareq Al Tell, Mohammad H Ashraf, Abdulaziz Al Failakawi, Qaisar I Khan, Ahmed M Nasar, James Lucocq

**Affiliations:** 1 Department of Otorhinolaryngology, Queen Elizabeth University Hospital, Glasgow, GBR; 2 Department of Trauma and Orthopaedics, Glasgow Royal Infirmary, Glasgow, GBR; 3 Department of Neurology, University of Glasgow, Glasgow, GBR; 4 Department of General Surgery, Sabah Hospital, Kuwait, KWT; 5 Department of Medical Education, University of Aberdeen, Aberdeen, GBR; 6 Department of Medical Education, University of Glasgow, Glasgow, GBR; 7 Department of General Surgery, Queen Elizabeth University Hospital, Glasgow, GBR; 8 Department of Trauma and Orthopaedics, Queen Elizabeth University Hospital, Glasgow, GBR; 9 Department of General Surgery, Victoria Hospital, Kirkcaldy, GBR

**Keywords:** resource-poor setting, mortality rate in sepsis, health education & awareness, surviving sepsis guidelines, global health policy

## Abstract

Introduction

Despite significant advances in the field of medicine, sepsis is constantly growing as a major public health concern. The global epidemic of sepsis imposes a significant economic burden on healthcare systems world-over. Furthermore, its high prevalence in society is inevitably paralleled by an excessive mortality rate, with approximately six million deaths reported every year. The primary aim of this study was to evaluate and compare, the management of acutely septic patients against outcomes in a tertiary teaching institution in Pakistan versus a similar one in the United Kingdom.

Methods

This study was a dual-centred, retrospective comparative analysis comparing all patients admitted through the emergency department at the respective tertiary centres. Patient details were collected and compared across the two sites to evaluate the effect of individual characteristics on prognosis. The outcomes of these presentations were analysed by comparing rates of in-hospital mortality, admission to the ICU or discharge.

Results

The total number of patients identified as having sepsis was 60 in the Pakistan cohort, and 92 in the Aberdeen cohort. No significant difference was found when comparing genders, and the results of basic observations were largely similar at presentation. Twenty-five per cent (25%) (n=38) of the total study population were deemed to have a poor outcome at 3 days, but 50% of the Pakistan cohort was deemed to have a poor outcome.

Conclusion

Managing sepsis has developed significantly in recent years, but most of this development was implemented in high-income countries. There was a significant delay in time to resuscitate septic patients in Pakistan, with significantly raised three-day morbidity and mortality. There is a need for further comparative studies of the management of sepsis in Pakistan and other low-income countries to identify the problems and tackle obstacles on every level of the healthcare system.

## Introduction

Despite significant advances in the field of medicine, sepsis is constantly growing as a major public health concern. The global epidemic of sepsis imposes a significant economic burden on healthcare systems world-over. Furthermore, its high prevalence in society is inevitably paralleled by an excessive mortality rate, with approximately six million deaths reported globally, every year [1,2]. The high mortality rate attributed to sepsis, along with the accumulating epidemiological evidence suggesting its continuation to dominate morbidity trends in the future, poses a significant challenge to all healthcare systems. This requires an influx of adequate resources and necessitates timely treatment to prevent further complications and subsequent death [1,3-5].

The World Health Organization defines sepsis as “a life-threatening organ dysfunction caused by a dysregulated host response to infection” [2,6]. Septic shock may develop if coupled with elevated lactate levels or hypotension [7,8]. Causes of sepsis are known to be multifactorial but can include any infectious agent that can trigger the abnormal host response. The causative pathogen must multiply in the bloodstream for sepsis to develop. This is why sepsis originates because of a physical or immunological breach of the integrity of the host barrier and the direct penetration of the pathogen into the bloodstream [9].

The clinical spectrum of sepsis is widely variable. Signs and symptoms can range from fever, localised pain and abdominal discomfort to hypotension and altered mental status. This is dependent on numerous factors, including the type of the causative pathogen, the portal of entry and the host susceptibility, among other factors. Because the disease may include a wide range of possible presentations, setting a classical concept may not necessarily be appropriate [8-10]

For a diagnosis of sepsis under the Systemic Inflammatory Response Syndrome (SIRS) criteria, two or more of the following findings should be confirmed [10]: 1. A body temperature >38°C or <36°C; 2. A heart rate over 90 beats/minute; 3. Respiratory rate >20 breaths/minute or arterial CO2 tension <32 mmHg; 4. White blood cell count >12,000/ mm^3^ or <4000/mm^3^.

The quick Sequential Organ Failure Assessment (qSOFA) is significantly predictive of increased all-cause mortality in patients outside of the ICU when the score is ≥2. The score assesses the following parameters: 1. Respiratory rate ≥22 breaths per minute; 2. Altered mentation/ Glasgow Coma Scale (GCS) <15; 3. Systolic blood pressure (SBP) <100 mmHg.

In the UK, over 48,000 deaths are attributed to sepsis annually, with this number rising each year [11]. A statistic is unfortunately not available in Pakistan, but in their international audit, Vincent et al. estimated that the burden of sepsis and mortality rates related to sepsis were much higher in lower-income countries in Asia and Africa [4].

The primary aim of this study was to evaluate and compare the management of acutely septic patients against outcomes in a tertiary teaching institution in Pakistan versus a similar one in the United Kingdom. Secondarily, this study reviews the use of scores such as the qSOFA and Shock Index (SI) in predicting morbidity and mortality in developing nations and thirdly investigates the causes of these differences. To the best of our knowledge, this is the first study that compares sepsis management between Pakistan and the UK.

## Materials and methods

This study was a dual-centred, retrospective comparative analysis comparing all patients admitted through the emergency department at the respective tertiary centres. The data were collected over a one-month period. The diagnosis of sepsis was based on the clinician's diagnosis using the SIRS criteria. The case notes of these patients were then used to gather the relevant data, and to follow up with the patient for three days from their initial presentation to the emergency department.

The data were collected from a large urban tertiary hospital in Lahore, Pakistan, and compared to a similar cohort of patients in Aberdeen Royal Infirmary. Data were gathered prospectively from patients presenting to the emergency department as well as retrospectively for in-patients in the other units within both hospitals.

The diagnosis of sepsis was confirmed by clinicians based on the SIRS criteria detailed previously, after which the case notes of these patients were analysed to gather the relevant data. Patients identified in the emergency department were observed whilst receiving treatment and followed up in their ward of admission for three days.

Patient data were collected and compared across the two sites to evaluate the effect of individual characteristics (age and location) on the prognosis. The measured parameters used to evaluate the prognosis of these septic patients are included in Table 1.

**Table 1:** Sepsis identification and management parameters

Data collected for septic patients in both cohorts:
Initial observations: Temperature, Respiratory Rate, Heart Rate, Blood Pressure, Oxygen Saturation, Glasgow Coma Scale
Time taken for administration of antibiotics
Time taken for administration of intravenous fluids
Time taken for administration of oxygen
The outcome of patients at three days

Following this, the outcomes of these presentations were analysed by comparing rates of in-hospital mortality, admission to ICU or discharge so that a conclusion regarding the influence of each of these patient details could be drawn. Results were analysed using the STATA 17.1 software (StataCorp. 2021. Stata Statistical Software: Release 17. College Station, TX: StataCorp LLC.). Comparative analysis was conducted using the chi-squared and Mann-Whitney U tests.

## Results

The total number of patients identified as having sepsis was 60 in the Pakistan cohort, and 92 in the Aberdeen cohort. For the purposes of this study, admission to ICU or death three days post-presentation was considered a poor outcome. Table 2 shows the overall characteristics with the comparison of the patients at presentation against a poor outcome.

**Table 2:** Overall characteristics of the study population 1. Median (IQR); n (%) 2. Wilcoxon rank sum test; Pearson's chi-squared test; Fisher's exact test

Characteristics	Overall, N = 152_1_	Aberdeen, N = 92_1_	Lahore, N = 60_1_	p-value_2_
Age	64 (41, 76)	70 (57, 79)	46 (34, 67)	<0.001
Gender (Male/Female)	97 (64%) / 55 (36%)	57 (62%) / 35 (38%)	40 (67%) / 20 (33%)	0.6
Temperature (°C)	38.10 (37.70, 38.55)	38.20 (37.25, 38.85)	38.10 (37.98, 38.40)	>0.9
Respiratory Rate (breaths per minute)	22.0 (18.0, 25.0)	20.0 (18.0, 24.0)	24.0 (20.8, 26.0)	<0.001
Systolic BP (mmHg)	120 (109, 139)	132 (113, 145)	116 (107, 123)	<0.001
Heart Rate (beats per minute)	102 (90, 112)	106 (92, 116)	98 (90, 106)	0.025
Oxygen Saturation (%)	95.0 (92.0, 97.0)	96.0 (94.0, 98.0)	93.0 (91.0, 95.0)	0.002
SI	0.82 (0.71, 0.98)	0.81 (0.67, 0.94)	0.88 (0.75, 1.00)	0.11
qSOFA Scores:				<0.001
0	7 (4.6%)	0 (0%)	7 (12%)	
1	71 (47%)	37 (40%)	34 (57%)	
2	70 (46%)	55 (60%)	15 (25%)	
3	4 (2.6%)	0 (0%)	4 (6.7%)	

The median age of patients across the two sites differed greatly, with Aberdeen having a median age of 70 and Pakistan having a much lower age of 46. No significant difference was found when comparing genders and the results of basic observations were largely similar at presentation. No significant difference was found when comparing shock index across the two sites at presentation however, the qSOFA scores varied, with a larger proportion of patients in Aberdeen having a score of two, while most patients in Pakistan had a score of 1. Additionally, only patients in Pakistan had a qSOFA score of 3, with these patients making up 6.7% of the cohort.

The management of these patients was assessed by measuring the time taken to administer antibiotics and intravenous fluids following presentation to the hospital. The outcomes were assessed by recording the outcome of the patient 72 hours after their initial presentation to the hospital and would include ward admission, intensive care unit (ICU) admission or in-hospital mortality. All these assessments are shown in Table 3.

**Table 3:** Comparison of management and outcomes of the two cohorts 1. n (%) 2. Pearson's chi-squared test

Characteristics	Overall, N = 152_1_	Aberdeen, N = 92_1_	Lahore, N = 60_1_	p-value_2_
In-hospital mortality	24 (16%)	8 (8.7%)	16 (27%)	0.003
Death or ICU Stay >3 Days	38 (25%)	8 (8.7%)	30 (50%)	<0.001
IV antibiotics started in 0-59 minutes	86 (57%)	85 (92%)	1 (1.7%)	<0.001
IV antibiotics started in more than 1 hour	66 (43%)	7 (7.6%)	59 (98.3%)	<0.001
IV fluids commenced in 0-59 minutes	87 (58%)	87 (95%)	0 (0%)	<0.001
IV fluids commenced in more than 1h	64 (42%)	5 (5.4%)	59 (98.3%)	<0.001

Twenty-five per cent (25%; n=38) of the total study population were deemed to have a poor outcome at three days, but 50% of the Pakistan cohort were deemed to have a poor outcome. Of those 50% (n=30), a further 50% (n=15) remained in the ICU for three days, and the remainder died at three days.

## Discussion

The overall aim of this study was to evaluate the management of sepsis in a tertiary teaching hospital in Pakistan and evaluate the short-term outcomes in a similarly sized hospital in the United Kingdom. The vast incidence of sepsis throughout the world increases the necessity for this study to identify and address any gaps in the clinical management of septic patients, particularly in developing nations.

Managing sepsis has developed significantly in recent years, but most of this development was implemented in high-income countries, which was reflected in reduced mortality and morbidity resulting from this condition [12,13]. While low-income countries still carry most of the burden of this serious medical problem, with no or rare efforts to change the practice toward this condition at an organisational level [2,3,5]. Our results confirm this observation, as this study found that there was a significantly increased risk of poor outcomes in the Pakistan cohort, with 50% of the patients dying or being admitted to ICU in comparison to just 8.7% (n=8) of the Aberdeen cohort.

This study found that there was no statistically significant difference between males and females, SI, and patient temperature on admission. However, respiratory rate, systolic blood pressure, heart rate, oxygen saturation and qSOFA score all showed significant differences between the two cohorts. The patient average age in the Pakistan group was lower, which can be understood in the context of the younger community in Pakistan compared to that of the United Kingdom.

The delay in time to resuscitate septic patients in the Pakistan cohort can be attributed to multiple factors: delayed recognition of sepsis due to the lack of diagnostic triage protocol for severely ill patients and the subsequent delay in alerting systems for doctors and nurses to attend this patient and start the treatment as soon as possible. Asiime et al. have shown a direct correlation between mortality rates in septic patients and the lack of such protocols that regulate recognition and intervals of vital signs monitoring in a low-resource environment [14]. Secondly, the educational gap that emphasises the importance of early resuscitation in reducing poor outcomes is shown by the lack of treatment guidelines and awareness campaigns for healthcare workers. These factors can be seen as well in other lower-income countries in Africa, Asia and Brazil, where reports and studies showed similar patterns of less optimised sepsis management and poor outcomes [15-17].

On the other hand, the Surviving Sepsis Campaign that was run in Scotland and the UK from 2002-2008, and the subsequent changes in the local guidelines and protocols to recognise and treat sepsis promptly have shown a huge effect on reducing mortality and morbidity as shown in the management of sepsis in Scotland report in 2010 by the Scottish Trauma Audit Group [12], where early recognition and early resuscitation played a huge role in reducing the burden and effects of this condition. The Surviving Sepsis Campaign advocated a bundle titled ‘Sepsis Six’, which comprised six steps to be delivered within one hour of reaching a diagnosis of sepsis [18,19]. These are detailed in Table 4.

**Table 4:** Sepsis six bundle

'Sepsis Six' bundle for managing sepsis in the first hour of the patient presentation
1. Titrate oxygen as required
2. Take blood cultures
3. Administer empirical intravenous broad-spectrum antibiotics
4. Measure serum lactate
5. Administer intravenous fluid for resuscitation
6. Accurate urine output measurement

The study has multiple limitations: The follow-up was only for three days and further follow-up until discharge can help understand the disease's effects on patients and institutes. Secondly, there was not enough data on the causes of the delay in providing treatment for patients in the Lahore cohort, as this would help understand the defects in the sepsis management in these environments and guide any further changes that may be needed. Increasing the sample size and involvement of more hospitals in the study may allow for a deeper understanding of the comparative picture between two cohorts and between high-income countries versus low-income countries.

## Conclusions

This study emphasises that managing sepsis in low-income countries remains deficient and needs massive improvements on all levels of the healthcare hierarchy. Furthermore, it shows the impact of having clear guidelines and algorithms on the management of patients presenting with sepsis when compared to the absence of such guidance. Additionally, this shows the huge role of education and healthcare professional awareness to improve the outcomes of these patients.

It is recommended that the management of sepsis in Pakistan and other low-income countries requires further comparative studies, and an objective systematic approach should be used to identify the problems and tackle obstacles at every level of the healthcare system. This would require a combination of efforts by local and international organisations to improve the current medical practice regarding sepsis.
